# Proton pump inhibitors protect mice from acute systemic inflammation and induce long-term cross-tolerance

**DOI:** 10.1038/cddis.2016.218

**Published:** 2016-07-21

**Authors:** E Balza, P Piccioli, S Carta, R Lavieri, M Gattorno, C Semino, P Castellani, A Rubartelli

**Affiliations:** 1 Cell Biology Unit, IRCCS AOU San Martino-IST, 16132 Genoa, Italy; 2 Pediatrics II Unit, G Gaslini Institute, 16147 Genoa, Italy; 3 Protein Transport Unit, Division of Cell and Molecular Biology, San Raffaele Institute, 20132 Milan, Italy

## Abstract

Incidence of sepsis is increasing, representing a tremendous burden for health-care systems. Death in acute sepsis is attributed to hyperinflammatory responses, but the underlying mechanisms are still unclear. We report here that proton pump inhibitors (PPIs), which block gastric acid secretion, selectively inhibited tumor necrosis factor-*α* (TNF-*α*) and interleukin-1*β* (IL-1*β*) secretion by Toll-like receptor (TLR)-activated human monocytes *in vitro*, in the absence of toxic effects. Remarkably, the oversecretion of IL-1*β* that represents a hallmark of monocytes from patients affected by cryopyrin-associated periodic syndrome is also blocked. Based on these propaedeutic experiments, we tested the effects of high doses of PPIs *in vivo* in the mouse model of endotoxic shock. Our data show that a single administration of PPI protected mice from death (60% survival *versus* 5% of untreated mice) and decreased TNF-*α* and IL-1*β* systemic production. PPIs were efficacious even when administered after lipopolysaccharide (LPS) injection. PPI-treated mice that survived developed a long-term cross-tolerance, becoming resistant to LPS- and zymosan-induced sepsis. *In vitro*, their macrophages displayed impaired TNF-*α* and IL-1*β* to different TLR ligands. PPIs also prevented sodium thioglycollate-induced peritoneal inflammation, indicating their efficacy also in a non-infectious setting independent of TLR stimulation. Lack of toxicity and therapeutic effectiveness make PPIs promising new drugs against sepsis and other severe inflammatory conditions.

Systemic inflammatory response is a critical clinical response to insults of either infectious or non-infectious origin.^1^ Severe sepsis and septic shock are more serious clinical forms with a poor outcome.^2^ The incidence of sepsis is continuously increasing;^1, 2, 3, 4^ the mortality rate ranges between 30 and 50% in severe sepsis and septic shock, and patients who survive have a higher risk of mortality compared with the normal population for months and even years.^5^ Although treatment of the underlying infection and circulatory support decrease mortality, sepsis remains a leading cause of death in critically ill patients, and efficacious therapy is missing.^6^

Traditionally, the physiopathology of sepsis is attributed to a hyperinflammatory response, the ‘cytokine storm', that can directly lead to death or favor the insurgence of an immunosuppressive phase during which multiple organ dysfunction occurs.^1^ We have recently reproduced *in vitro* on primary monocytes the cytokine storm: the simultaneous activation of multiple Toll-like receptors (TLRs) results in oxidative stress responsible for a marked enhancement of tumor necrosis factor-*α* (TNF-*α*) and interleukin-1*β* (IL-1*β*) secretion.^7^

Proinflammatory cytokines are indeed increased in sepsis. TNF-*α*^8^ is the first cytokine detected in the serum of septic patients followed by IL-1*β*.^9^ High-mobility group box chromosomal protein-1 appears well after TNF-*α* and IL-1*β*, supporting its role in mortality due to late sepsis.^10^

The ‘cytokine storm' theory is presently debated, mainly because clinical trials with cytokine antagonists were unsuccessful.^11^ Timing of administration and non-optimal association of cytokine-neutralizing agents may be responsible for the failure of clinical trials; however, other factors besides cytokine hypersecretion, including genetic polymorphism in genes for coagulation and fibrinolysis and the insurgence of oxidative stress,^2^ may participate in the genesis of sepsis. A common trait in septic patients is acidosis, which is more pronounced in non-survivors.^12^ Acidosis is mainly due to a shift from oxidative phosphorylation to glycolysis, with accumulation of lactate and decrease of pH,^13^ and occurs upstream of the pathologic events (release of toxic substances, vasoconstriction, endothelial damage) that lead to cell and patient death.^12^ Acidosis is also a feature of inflammatory microenvironments:^14, 15^ upon activation, inflammatory cell metabolism shifts toward aerobic glycolysis,^16^ with consequent decrease of extracellular pH. Extracellular acidosis is proinflammatory: it induces inflammatory genes^14, 15^ and increases processing and secretion of IL-1*β*^17^ in a NOD-like receptor (NLR) family, pyrin domain containing 3 (NLRP3) inflammasome-dependent^18, 19^ or -independent^20^ manner.

Proton pump inhibitors (PPIs) are a family of prodrugs that, activated by low pH,^21^ are highly efficacious in reducing acidic secretion by gastric cells and therefore largely used in the treatment of peptic ulcers and reflux esophagitis.^22^ PPIs are also effective against tumors, which are similarly characterized by low pH.^23, 24^ Furthermore, PPIs have been found to exert anti-inflammatory effects unrelated to the inhibition of gastric acid production, although the underlying mechanisms remain to be elucidated.^25^

Here we show that, *in vitro*, PPIs inhibit the production of proinflammatory cytokines by monocytes stimulated with TLR agonists; *in vivo*, in a murine model of lethal endotoxic shock,^8, 26^ PPIs protect against lipopolysaccharide (LPS)-induced mortality. Interestingly, mice cured by PPIs develop cross-tolerance: not only are they resistant to a second challenge with LPS but also respond better to zymosan injection.

## Results

### Inhibitory effect of PPIs on PAMP-induced IL-1*β* and TNF-*α* secretion *in vitro*

**Figure 1:**
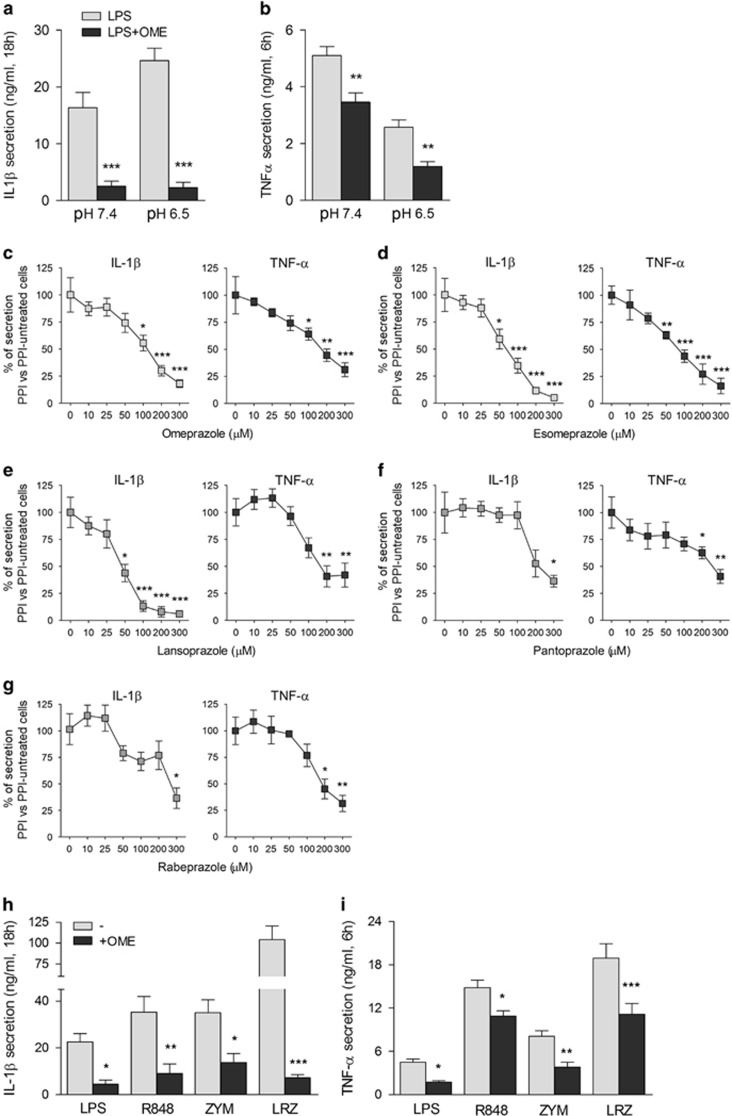
OME inhibits IL-1*β* and TNF-*α* secretion induced by different PAMPs in human healthy monocytes. (**a** and **b**) Healthy monocytes were incubated in the medium at neutral pH (pH 7.4) or acidic pH (pH 6.5) with LPS (100 ng/ml) in the absence or presence of OME (300 *μ*M). Secreted IL-1*β* (**a**) and TNF-*α* (**b**) were quantified after 18 and 6 h, respectively. Data are expressed as ng/ml (*N*=5, mean±S.E.M.). (**c**–**g**) Dose–response experiments with 10–300 *μ*M of OME (**c**), ESO (**d**), lansoprazole (**e**), pantoprazole (**f**) and rabeprazole (**g**) were performed. Supernatants were collected after 18 or 6 h to quantify IL-1*β* (left panels) and TNF-*α* (right panels). Data are expressed as the percentage of secretion of PPI *versus* PPI-untreated cells; mean±S.E.M. of four experiments. (**h** and **i**) Monocytes were stimulated for 18 and 6 h with LPS (100 ng/ml), R848 (5 *μ*g/ml) and zymosan (ZYM, 20 *μ*g/ml), alone or in combination (LRZ), in the presence or absence of OME. Secreted IL-1*β* (**h**) and TNF-*α* (**i**) were quantified as above. Data are expressed as ng/ml (*N*=5, mean±S.E.M.). *P<0.05; **P<0.01; ***P<0.001

Secretion of IL-1*β* by primary human monocytes activated with LPS was increased at low pH (Figure 1a), in agreement with the previous data.^17, 18, 19, 20^ Interestingly, IL-1*β* secretion was strongly inhibited by the PPI omeprazole (OME) both at acidic and neutral pH (Figure 1a). OME displayed an IC_50_ of 100 *μ*M, and inhibited IL-1*β* secretion up to 80% at 300 *μ*M (Figure 1c, left panel). Although LPS-induced TNF-*α* is not increased by low pH, OME also inhibited TNF-*α* secretion (Figures 1b and c, right panel). Dose–response experiments with other PPIs^21, 22^ provided data similar to OME both for IL-1*β* and TNF-*α* (Figures 1d–g). Toxicity, evaluated by trypan blue staining and lactate dehydrogenase release, was virtually absent at doses lower than 400 *μ*M with all PPIs tested (not shown). The inhibitory effect of PPIs on cytokine production is not restricted to TLR4 stimulation: also R848- (TLR7/8 ligand) and zymosan- (TLR2 ligand) induced secretion of IL-1*β* and TNF-*α* was impaired (Figures 1h and i). Similarly, the marked secretion of IL-1*β* and TNF-*α* that follows the simultaneous stimulation of monocytes with the three TLR ligands^7^ was inhibited (Figures 1h and i; LRZ).

**Figure 2:**
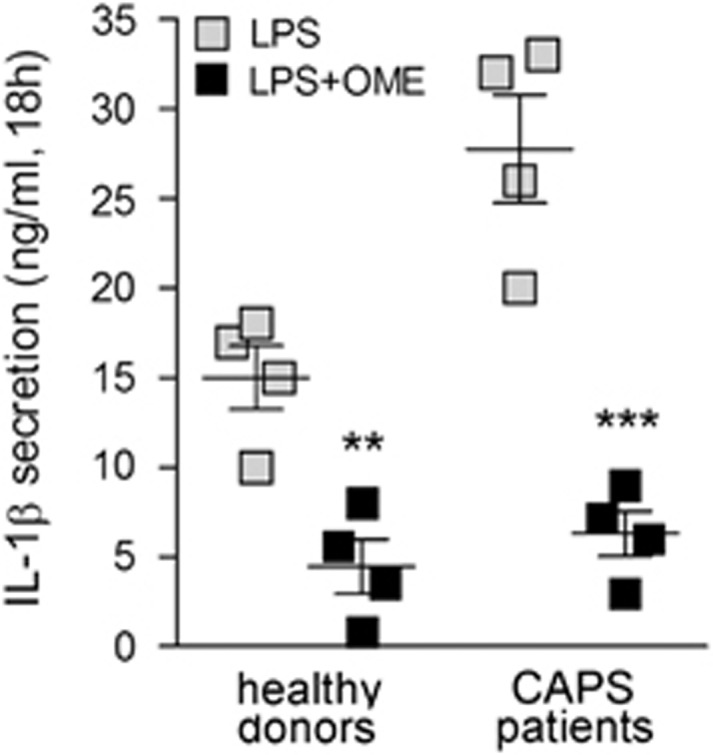
OME prevents secretion by monocytes from patients affected by CAPS. Monocytes from CAPS patients (*N*=4) and healthy donors (*N*=4) were stimulated with 100 ng/ml of LPS alone or in combination with OME (300 *μ*M). Secreted IL-1*β* was quantified by enzyme-linked immunosorbent assay (ELISA) in 18 h supernatants. Data are expressed as ng/ml. **P<0.01; ***P<0.001

The effect of OME on IL-1*β* secretion was investigated on monocytes from patients affected by cryopyrin-associated periodic syndrome (CAPS), a very rare autoinflammatory disease where gain-of-function mutations in the inflammasome gene NLRP3 cause huge secretion of IL-1*β*.^27^ As shown in Figure 2, OME inhibited IL-1*β* secretion by >80% in all the four patients examined.

### PPIs inhibit TNF-*α* and IL-1*β* secretion at different levels

**Figure 3:**
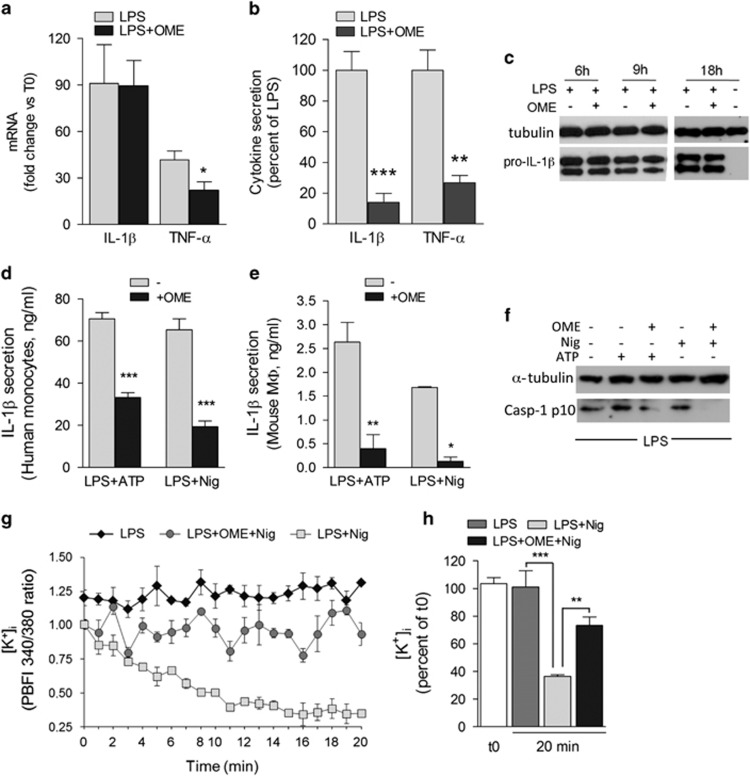
OME downmodulates IL-1*β* and TNF-*α* secretion with different mechanisms. (**a**) Real-time PCR of IL-1*β* and TNF-*α* mRNA levels, 3 h from exposure to LPS or LPS+OME. Data are expressed as fold change of mRNA levels in cells stimulated with LPS or LPS+OME *versus* untreated cells (mean of normalized expression±S.E.M.; *N*=4). (**b**) IL-1*β* (18 h) and TNF-*α* (6 h) secreted by monocytes from the same subjects analyzed in (**a**). Data are expressed as the percent of secretion by LPS+OME *versus* LPS (mean±S.E.M.). (**c**) Western blot analysis of intracellular pro-IL-1*β* in monocytes unstimulated or at different time points from LPS stimulation, with or without OME. *α*-Tubulin is used as the loading control. One representative experiment out of five is shown. (**d** and **e**) IL-1*β* secreted by human monocytes (**d**) or murine peritoneal macrophages (**e**) primed 3 h (**d**) or 18 h (**e**) with LPS and then exposed 30 min to ATP, at 1 mM (**d**) or 5 mM (**e**) or to 20 *μ*M nigericin (Nig, 20 min) with or without OME (mean±S.E.M.; *N*=4). (**f**) Western blot of p10 caspase-1 in cell lysates from murine macrophages. *α*-Tubulin is shown as the loading control (one representative experiment out of three). (**g**) Monocytes stimulated 3 h with LPS were loaded with PBFI and incubated in medium alone (LPS) or with 20 *μ*M nigericin without (LPS+Nig) or with 300 *μ*M OME (LPS+Nig+OME). Data are expressed as 340/380 ratio of the PBFI fluorescence intensity measured every 60 s for 20 min (mean±S.E.M.; *N*=3). (**h**) PBFI fluorescence intensity after 20 min in the different culture conditions depicted in (**g**) is expressed as percent *versus* time 0. *P<0.05, **P<0.01; ***P<0.001

The amount of TNF-*α* mRNA in monocytes stimulated with LPS in the presence of OME was found to be ~50% less than that detected in monocytes exposed to LPS alone (Figure 3a), a decrease consistent with the decreased TNF-*α* secretion (Figure 3b). However, no difference in the activation of inflammation-related transcription factors, such as nuclear factor-*κ*B (NF-*κ*B) and activator protein-1 (AP-1), was observed in untreated or OME-treated monocytes (Supplementary Figure S1). Moreover, in spite of the marked inhibition of secretion (Figure 3b), OME affected neither IL-1*β* gene expression (Figure 3a) nor intracellular accumulation of the precursor pro-IL-1*β* protein in LPS-stimulated monocytes (Figure 3c), suggesting that the inhibitory effect of the drug is located post-translationally, at the level of inflammasome activation. Accordingly, both on LPS-primed monocytes (Figure 3d) and peritoneal murine macrophages (Figure 3e), OME inhibited IL-1*β* secretion induced by extracellular adenosine triphosphate (ATP) or nigericin, a toxin that strongly activates the inflammasome,^28^ and prevented ATP- and nigericin-induced generation of active caspase-1 (Figure 3f). Both ATP and nigericin trigger a marked efflux of K^+^ from cells, resulting in a drop in [K^+^]_i_, which is a crucial step in NLRP3 inflammasome activation and IL-1*β* secretion.^29, 30^ Remarkably, nigericin-induced K^+^ release from monocytes was prevented by OME (Figures 3g and h). Thus, OME directly or indirectly interferes with K^+^ efflux, resulting in hindrance of inflammasome assembly and caspase-1 activation, and consequently of IL-1*β* secretion.

**Figure 4:**
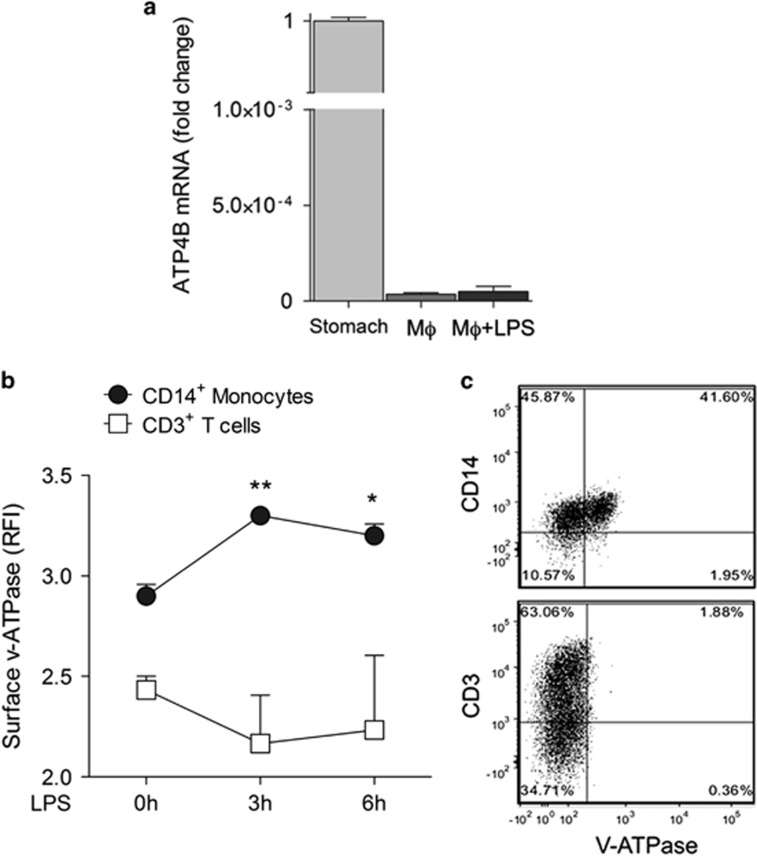
Macrophages do not express the gastric H^+^/K^+^ ATPase, but display surface v-ATPases. (**a**) Reverse transcription-polymerase chain reaction (RT-PCR) analysis of mRNA coding for the *β*-subunit of the gastric H^+^/K^+^ proton pump on peritoneal mouse macrophages (M*φ*) untreated or exposed 3 h to LPS (M*φ*+LPS). Data are expressed as fold changes of normalized expression *versus* murine stomach (mean±S.E.M. of three experiments). (**b** and **c**) PBMCs from healthy donors were double stained with anti-v-ATPase and anti-CD14 antibody (Ab) or anti-CD3 Ab time 0 or 3 or 6 h after exposure to LPS and analyzed by FACS. In (**b**), data are expressed as the relative fluorescence intensity (RFI) of v-ATPase in CD14^+^ (monocytes) and CD3^+^ (T-lymphocytes) cells (mean±S.E.M. of three independent experiments). Statistical analysis is referred to monocytes and evaluated *versus* *t*0. *P<0.05; **P<0.01 *versus* (**c**) a representative experiment of costaining (out of 3) is shown: 41% of CD14^+^ cells (upper plot) and 1.88% of CD3^+^ cells are positive for surface v-ATPases at 3 h from LPS exposure. *P<0.05; **P<0.01

The molecular target of PPIs on gastric cells is the hydrogen potassium ATPase (H^+^/K^+^ ATPase) proton pump.^21^ However, macrophages do not express this proton pump (Figure 4a). Among the other proton pumps, we focused on vacuolar ATPases (v-ATPases). These pumps, usually restricted to intracellular acidic organelles,^31^ are expressed on the surface of certain cell types including tumor cells where they were proposed to be a target of PPIs.^32^ Interestingly, CD14^+^ monocytes, unlike CD3^+^ lymphocytes, are positive for surface v-ATPases and positivity increases following LPS stimulation (Figures 4b and c), suggesting a possible role of surface-bound v-ATPases as PPI receptors on activated monocytes.

### Treatment with esomeprazole protects mice from LPS-induced sepsis

**Figure 5:**
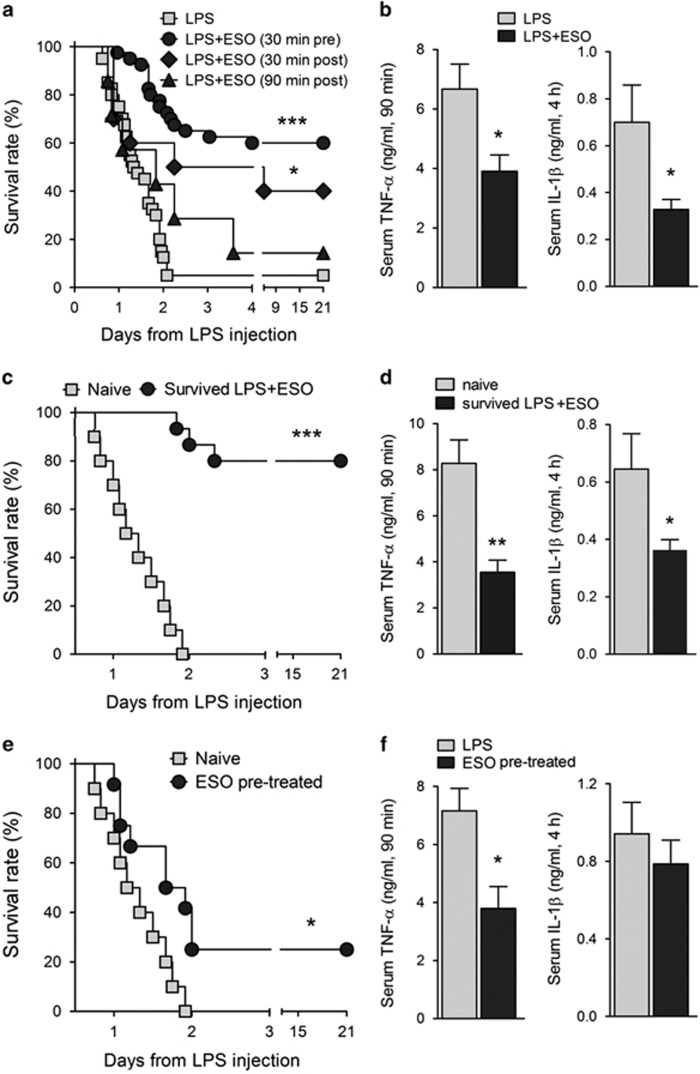
ESO protects mice from LPS shock, suppresses the systemic production of TNF-*α* and IL-1*β* and induces resistance to LPS rechallenge. (**a**) Mice were injected intravenously with LPS (12.5 mg/kg) alone (*N*=40), or received intraperitoneally ESO (12.5 mg/kg) 30 min before (*N*=40), 30 min after (*N*=10) or 90 min after (*N*=7) the LPS injection. Mice were monitored for survival. (**b**) TNF-*α* (left panel) and IL-1*β* (right panel) in sera from LPS- and LPS+ESO-treated mice were quantified (ng/ml) 90 min (TNF-*α*) or 4 h (IL-1*β*) after LPS injection, respectively (mean±S.E.M., *N*=11). (**c**) Fifteen mice ESO treated and that survived the first LPS shock (survived LPS+ESO) were rechallenged with LPS, without any treatment, 3 weeks after the first LPS injection. As control, 10 naive mice were injected with LPS. Mice were monitored for survival. (**d**) TNF-*α* (left) and IL-1*β* (right) levels (ng/ml) were detected in the serum of naive and rechallenged mice by enzyme-linked immunosorbent assay (ELISA) (mean±S.E.M.; *N*=8 for TNF-*α*, *N*=7 for IL-1*β*). (**e**) Twelve mice receive a single injection of ESO 15 days before LPS challenge (ESO pre-treated). A control group of 10 naive mice received LPS only. Mice were monitored for 21 days for survival. (**f**) Serum levels of TNF-*α* (left) or IL-1*β* (right) from ESO pre-treated and naive mice were quantified as above. Data are expressed as ng/ml (mean±S.E.M.; *n*=6). *P<0.05, **P<0.01 and ***P<0.001

The above results prompted us to investigate the therapeutic potentials of PPIs in systemic inflammation *in vivo*. Esomeprazole (ESO) was preferred to OME, as it was found superior in reducing gastric acid secretion when parenterally administered.^33^ A murine model of acute endotoxic shock was used.^26^ Forty mice were injected intravenously with a lethal dose of LPS (12.5 mg/kg); a second group of 40 mice received ESO intraperitoneally 30 min before LPS. In the first 24 h after LPS injection, mice from both groups exhibited signs of disease, including alterations in weight, temperature and mobility. Sixty percent of mice treated with ESO improved from the second day and fully recovered, whereas only two mice in the control group survived LPS injection (Figure 5a). The systemic production of TNF-*α* and IL-1*β* was strongly inhibited in mice treated with ESO (Figure 5b). At variance, the serum levels of other inflammatory and anti-inflammatory mediators including IL-10, IL-6 and IL-1 receptor antagonist (IL-1Ra) were not significantly changed in ESO-treated mice (Supplementary Figure S2). When ESO was injected 30 min after LPS injection, an important therapeutic effect was still evident, with 40% of survival (Figure 5a). Increase of survival time, with 14% of mice that fully recovered, was observed even when ESO treatment was performed after 1.5 h from LPS, when serum TNF-*α* had already reached a high level (Figure 5b).

### ESO-treated mice that survived shock are resistant to rechallenge with LPS

A group of 15 LPS+ESO-treated mice that recovered were rechallenged with LPS, without other treatments, at 3 weeks after the first injection. A control group of naive mice (*N*=10) received the same dose of LPS. Remarkably, 80% of mice that had recovered from the first shock (Figure 5c, survived LPS+ESO) also survived the rechallenge (Figure 5c) without exhibiting overt signs of disease. In contrast, all naive mice died. The systemic production of TNF-*α* and IL-1*β* after the second challenge of LPS was much lower in ESO-treated mice that survived the first LPS injection than in control mice (Figure 5d).

As a control, the potential suppressive effect of ESO *per se* was studied. Two groups of mice were injected with a single dose of ESO alone or with an equal volume of saline solution. After 2 weeks, both groups received a lethal dose of LPS (12.5 mg/kg): while all control mice died within 48 h, 25% of ESO-pre-treated mice survived (Figure 5e). Systemic production of TNF-*α* by ESO-pre-treated mice after challenge with LPS was decreased with respect to controls, whereas IL-1*β* decrease was not significant (Figure 5f).

### Macrophages from mice that survived LPS injection display tolerance to different TLR agonists *in vitro*

**Figure 6:**
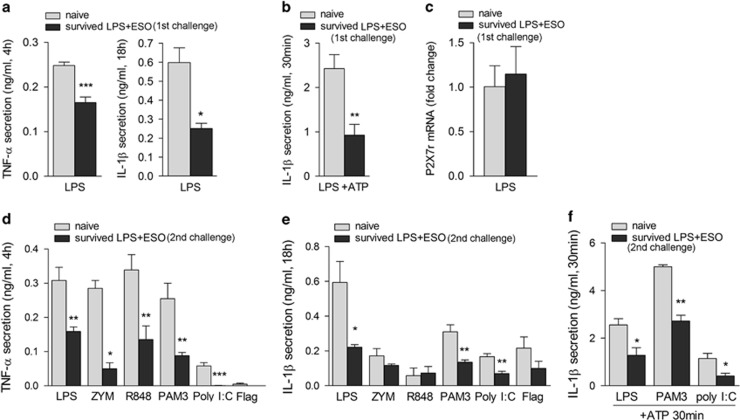
Macrophages from mice survived LPS rechallenge secrete less TNF-*α* and IL-1*β* in response to different TLR agonists. (**a** and **b**) Peritoneal macrophages from ESO-treated mice survived the first LPS shock (*N*=4) and from naive mice (*N*=6) were stimulated with LPS (**a**) or primed 18 h with LPS and then exposed 30 min to ATP (**b**), and TNF-*α* (**a**) and IL-1*β* (**a** and **b**) were quantified in supernatants after 4 or 18 h. (**c**) Real-time PCR analysis of P2X7 receptor mRNA from macrophages from naive mice or from mice survived the first LPS shock (survived LPS+ESO) after 3 h exposure to LPS. (**d–f**) Peritoneal macrophages from ESO-treated mice survived the second LPS shock (*N*=4) and from naive mice (*N*=6) were stimulated with LPS, zymosan (ZYM), R848, Pam(3)CSK(4) (PAM3), poly(I:C) and flagellin (Flag) for 4 (**d**) or 18 h (**e**) or with LPS, Pam(3)CSK(4) or poly(I:C) for 18 h followed by 30 min with 5 mM ATP (**f**). Secreted TNF-*α* (**d**) and IL-1*β* (**e** and **f**) were quantified (ng/ml; mean±S.E.M.; *N*=4). *P<0.05; **P<0.01; ***P<0.001

Peritoneal macrophages from ESO-treated mice that survived the first or the second LPS challenge were collected at 3 weeks after the LPS injection and stimulated *in vitro* with agonists of different TLRs (Figure 6). Significantly less TNF-*α* and IL-1*β* were secreted by macrophages from mice that survived compared with macrophages from naive mice in response not only to LPS (Figure 6a) but also to other TLR ligands (Figures 6d and e). Also, the burst of IL-1*β* secretion induced in primed macrophages by short exposure to ATP was strongly suppressed both in macrophages from mice that survived the first (Figure 6b) and the second LPS challenge (Figure 6e), in spite of an expression of the purinergic P2X (purinergic P2X, ligand-gated ion channel 7), ligand-gated ion channel 7 (P2X7) receptor similar to naive macrophages (Figure 6c).^34^ Suppression was long lasting: decreased cytokine production was still observed in macrophages collected from ESO-cured mice at 60 days after the first LPS injection (not shown).

The surface expression of v-ATPases was analyzed on macrophages from naive and ESO-cured mice at 3 weeks after LPS challenge. The expression was highly variable in the different mice examined, and similar in macrophages from naive and LPS+ESO-treated mice, either before or after LPS stimulation. However, a slightly less relative fluorescence intensity was observed in the macrophages from ESO-treated mice that survived LPS injection (Supplementary Figure S3).

### Mice that survived LPS-ESO display resistance to zymosan-induced generalized inflammation model

**Figure 7:**
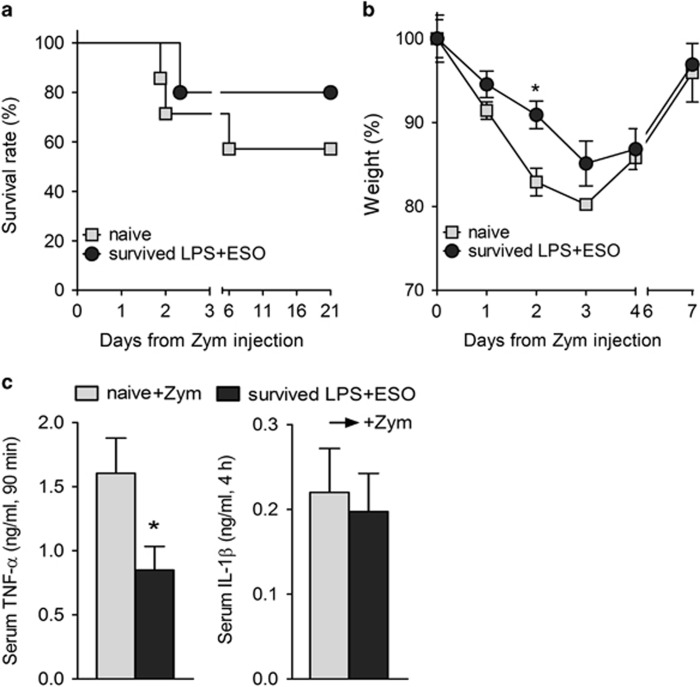
ESO-treated mice that survived LPS shock display resistance to zymosan-induced generalized inflammation. ESO-treated mice that recovered from the first LPS challenge (survived LPS+ESO, *N*=5) and naive mice (*N*=7) were injected with zymosan (Zym, 1 g/kg). Mice were monitored for survival (**a**) and for loss of body weight (**b**; *N*=4). (**c**) Serum levels of TNF-*α* (left; *N*=4 naive+Zym and *N*=5 survived Zym-treated mice) and of IL-1*β* (right; *N*=3 naive+Zym and *N*=4 survived Zym-treated mice) were quantified (ng/ml; mean±S.E.M.). *P<0.05

To understand whether the long-lasting suppressed cytokine production *in vitro* in response to different TLR agonists by macrophages from ESO-cured mice corresponds to a cross-resistance *in vivo*, an inducer of systemic inflammation other than LPS, namely, zymosan,^35^ was injected intraperitoneally in five ESO-treated mice that recovered from the first LPS challenge and in seven naive mice as control. Although this dose of zymosan was not lethal, resulting in 57% of mice that fully recovered, survival was higher in the group of mice that had survived LPS+ESO (80% Figure 7a). Moreover, among survivors, naive mice exhibited a significantly stronger weight loss in the first 2 days (Figure 7b), and more intense signs of disease. In mice that recovered from zymosan-induced inflammation, the serum level of TNF-*α* (but not of IL-1*β*) was significantly lower in the group of mice survived LPS+ESO than in naive mice (Figure 7c). Thus, ESO-treated mice that survived LPS shock acquire resistance also to zymosan.

### ESO is effective on thioglycollate-induced peritonitis

**Figure 8:**
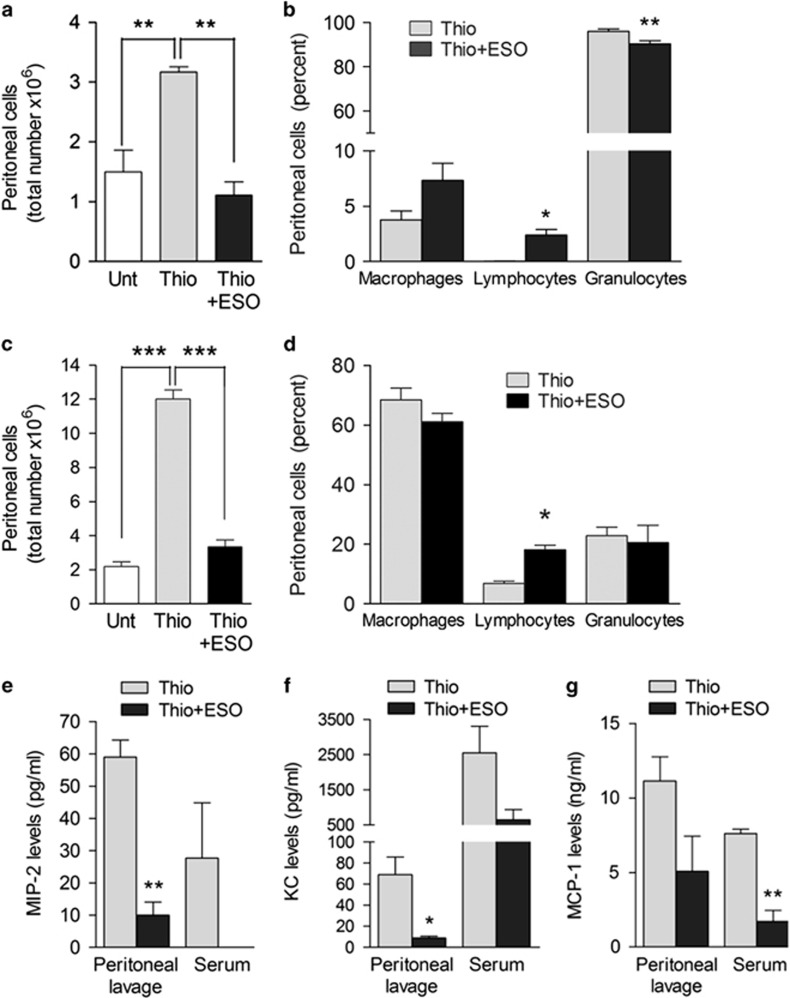
ESO prevents thioglycollate-induced peritonitis. (**a** and **c**) Peritoneal cells isolated 4 h (**a**) or 72 h (**c**) from untreated (Unt) mice or from mice injected intraperitoneally with thioglycollate alone (Thio) or thioglycollate 30 min after intraperitoneal injection with ESO (Thio+ESO) were counted. Data are expressed as the total number of infiltrating inflammatory cells (mean±S.E.M.; *N*=3). (**b** and **d**) The relative percent of macrophages, lymphocytes and granulocytes in peritoneum lavage from the same mice was calculated at 4 h (**b**) and 72 h (**d**). (**e**, **f** and **g**) MIP-2 (**e**), KC (**f**) and MCP-1 (**g**) levels were determined in serum and peritoneal lavage 4 h after thioglicollate treatment in untreated or ESO-treated mice (pg/ml; *N*=3, mean±S.E.M.) *P<0.05; **P<0.01; ***P<0.001

To address the efficacy of PPIs in a non-infectious setting independent of TLR stimulation, we used the sodium thioglycollate peritoneal inflammation mouse model.^36, 37^ Two groups of mice were injected intraperitoneally with thioglycollate and one of these received ESO intraperitoneally 30 min earlier. A third group received saline only. The number of leukocytes infiltrating the peritoneum of mice treated with ESO and thioglycollate was similar to that of control mice, and markedly lower compared with that of untreated, thioglycollate-injected mice, both after 4 (Figure 8a) and 72 h (Figure 8c). The percentage of infiltrating cells was instead similar, although, as expected,^37^ after 4 h neutrophils prevailed (Figure 8b), whereas after 72 h macrophages were the most represented cell type (Figure 8d). In keeping with these results, the levels of the neutrophil chemokines MIP-2 (macrophage inflammatory protein 2) and KC and of the monocyte chemoattractant protein-1 (MCP-1)^37^ were lower in peritoneal lavage and serum of ESO-treated mice compared with untreated, thioglycollate-injected mice (Figures 8e–g).

## Discussion

Despite many efforts, none of the newly developed drugs against sepsis has been translated into clinical settings.^6, 11^ This failure depends, in large part, on the complex and poorly understood pathophysiology of sepsis. Here we propose PPIs as effective drugs in severe sepsis or septic shock, based on the following pieces of evidence: (i) in the mouse model of acute lethal endotoxic shock, ~60% of mice treated with ESO 30 min before LPS injection undergo a complete recovery; (ii) ESO increases the survival rate even if administered after induction of shock; (iii) the systemic production of TNF-*α* and IL-1*β* is lowered in ESO-treated mice.

The last observation demonstrates that an important effect of PPI in sepsis is the limitation of the early cytokine storm that may lead to multiple organ dysfunction and death. The decrease of systemic TNF-*α* is stronger than that of IL-1*β*, in spite of both cytokines being similarly suppressed in macrophages from ESO-treated mice that survived shock. This is likely due to the fact that IL-1 activity is mainly exerted at the local level,^38^ and circulating IL-1*β* is lower compared with TNF-*α* in most inflammatory conditions, including sepsis.^39^ However, the finding that PPIs provide protection against sepsis even if administered after LPS injection, when the peak of TNF-*α* has already been reached and the cytokine starts to decrease, indicates that TNF-*α* is not the only target of PPI treatment and is not the only culprit in sepsis, coherently with the clinical data reporting the therapeutic failure of anti-TNF-*α* agents.^40^ The efficacy of PPIs injected when endotoxic shock is already developing is of considerable clinical importance, as a major critical point in curing severe sepsis is that patients must be treated very early after diagnosis since even a slight delay results in loss of any therapeutic effects.^41^

Our *in vitro* data on primary human monocytes show that PPIs are strong inhibitors of TNF-*α* and IL-1*β* secretion induced by different pathogen-associated molecular patterns (PAMPs), present on Gram-negative and -positive bacteria, viruses and yeast.^42^ The inhibitory effect of PPIs is also evident on monocytes simultaneously stimulated with various TLR agonists, a condition that mimics *in vitro* a cytokine storm, with huge increase of proinflammatory cytokine release.^7^ Thus, PPIs counteract inflammation induced by several different pathogens. Downmodulation of IL-1*β* secretion by PPIs occurs post-translationally, with block of K^+^ efflux and consequent inhibition of NLRP3 inflammasome activation. That NLRP3 is a main target of PPI action is supported by the strong inhibition of IL-1*β* secretion by monocytes from CAPS patients, carrying hypersensitive NLRP3 mutations, suggesting the potential use of PPIs also for the treatment of autoinflammatory syndromes.^27^ Unlike the recently described NLRP3 inhibitor MCC950,^43^ PPIs have a wider anti-inflammatory activity as they also inhibit TNF-*α* secretion, by decreasing its mRNA levels. Further studies are required to clarify the mechanism(s) underlying the decreased mRNA.

As we demonstrate that macrophages lack the H^+^/K^+^ ATPase proton pump targeted by PPIs on gastric cells,^44^ PPIs are likely to interact with a different, so far unidentified molecular target on monocytes/macrophages. Activated monocytes/macrophages undergo a shift to aerobic glycolysis, resulting in the generation of H^+^ that must be extruded to allow cell survival.^16^ Interestingly, we also observed that monocytes express on their surface v-ATPases that increase with LPS stimulation and that may mediate H^+^ externalization.^31^ An appealing possibility is that, as proposed for tumors,^32^ membrane-bound v-ATPases are blocked by PPIs, preventing H^+^ release and increasing the extracellular pH, thus reducing the acidosis of septic patients and its detrimental effects.^12^

A further important finding of our study is that ESO-treated mice that survived the endotoxic shock develop a long-lasting resistance (tested up to 2 months after the first sepsis) to a second LPS-induced sepsis. Although the first LPS challenge caused symptoms of shock in the first 12 h followed by a full recovery, LPS-rechallenged mice did not show signs of illness. Moreover, mice that survived LPS+ESO are more resistant than naive mice to a challenge with a different TLR agonist, such as zymosan.^35^ In all cases, the resistance to a second challenge (LPS or zymosan) was accompanied by a reduced systemic production of TNF-*α* and IL-1*β*. Thus, PPIs allow the generation of a nonspecific hyporesponsiveness similar to LPS-induced tolerance,^45, 46^ as confirmed by the finding that macrophages from ESO-treated mice that survived LPS shock display an impaired production of IL-1*β* and TNF-*α* after stimulation with several different TLR agonists in *ex vivo* experiments.

ESO displays a tolerizing effect *per se*, providing a partial protection to LPS-induced death when injected 2 weeks before LPS. In line with the lack of side effects in humans treated intravenously with high dosages of ESO in a single administration,^33, 47, 48^ mice displayed no adverse effects despite the dose used corresponded to 2–5 times the maximal doses given intravenously in human studies.^47, 48^ PPIs, at lower doses, are currently used for the stress ulcer prophylaxis in critically ill patients:^49^ our data, together with the studies on humans,^33, 47, 48^ suggest that systemic administration of higher doses of ESO in patients at risk of sepsis may help preventing the development of the syndrome without harmful side effects.

We also report that a single injection of ESO almost completely prevents the thioglycollate-elicited peritoneal infiltrate of inflammatory cells. As thioglycollate peritonitis is triggered by non-enzymatic reactions between proteins and reducing sugars leading to the formation of advanced glycation end-products recognized by advanced glycation end-product receptors,^36^ this result implies that PPIs are therapeutic on various types of inflammation, including sterile inflammation, independent of TLR. Remarkably, treatment with ESO strongly suppresses the production of MIP-2, KC and MCP-1 chemokines, which recruit neutrophils and macrophages following thioglycollate injection.^37^ As these chemokines are produced by neutrophils, this observation suggests that also these cells are target of PPIs.

Finally, PPIs have great advantages over the other newly developed drugs proposed for the treatment of sepsis: they are safe, displaying a very low risk of adverse events, which is almost absent in the case of a short-term use,^47^ and, as shown in this study, they are active against various noxiae. Importantly, they are low priced, available in most countries and among the most widely prescribed drug in the world.

For all these reasons, our study opens up an important new opportunity for the treatment of sepsis and septic shock due to infections with a wide variety of pathogens.

## Materials and Methods

### Chemicals and PAMPs

PIPES, MOPS, TES, HEPES, LPS (from *Escherichia coli* serotype 0111:B4), poly(I:C), ATP, zymosan, OME, ESO, lansoprazole, pantoprazole and rabeprazole were from Sigma-Aldrich (Milan, Italy); R848 and Pam(3)CSK(4) were from Invivogen (Milano, Italy); Flagellin was from Alexis Biochemicals (Enzo Life, Rome, Italy); nigericin was from Calbiochem (DBA, Milan, Italy); PBFI-aM (the cell-permeant acetoxymethyl ester of PBFI) and pluronic F-127 (a non-ionic detergent polyol used to facilitate cell loading of large dye molecules) were from Molecular Probes (Thermofisher, Monza, Italy).

### Human monocyte isolation and culture

Monocytes from healthy donors were cultured in RPMI-1640 with 5% FCS (Euroclone, Pero, MI, Italy) at 37 °C, 5% CO_2_ and stimulated with LPS (100 ng/ml), R848 (5 *μ*g/ml) and zymosan (20 *μ*g/ml) alone or in combination.^7^ Dose–response experiments were performed with all PPIs (Figure 1). OME was used in all the *in vitro* experiments at 300 *μ*M. When indicated, monocytes were primed 3 h with LPS followed by ATP (1 mM) or nigericin (20 *μ*M) for 20 min. Media at different controlled pH were carried out using bicarbonate-free RPMI-1640 (Sigma-Aldrich), buffered with 10 mM PIPES, 10 mM MOPS, 10 mM NaH_2_PO_4_ for pH 6.5; and with 10 mM TES, 10 mM MOPS, 15 mM HEPES, 2 mM NaH_2_PO_4_, for pH 7.4, as described.^17^ The experiments with medium at controlled pH were conducted at 37 °C in a non-CO_2_ incubator.

### Patients and blood samples

Four CAPS patients positive for mutations of the *NLPR3* gene and four age-matched healthy controls were studied in parallel. Blood samples were taken after informed consent by patients or parents, approved by the ‘G Gaslini' Ethical Board,^50^ and monocytes stimulated as above.

### Determination of intracellular K^+^ concentration

Intracellular K^+^ was determined using the K^+^-sensitive fluorophore PBFI.^29^ Human monocytes were seeded into 96-well culture plates at 7 × 10^5^ cells per well, in triplicate and stimulated with LPS in a potassium-free medium. After 90 min, cells were loaded with 5 μM PBFI-AM together with 0.05% (w/w) pluronic F-127 and incubated in the dark at 37 °C, for further 90 min, according to the manufacturer's instructions. After two washes in PBS, cells were cultured in potassium-free medium with or without nigericin (40 *μ*M) or nigericin plus OME (300 *μ*M) and fluorescence was immediately recorded every 60 s for 20 min, using a microplate fluorimeter set at 37 °C. Excitation wavelength was set at 340 and 380 nm, the isosbestic point. Emission was measured at 500 nm. The ratio of the fluorescence intensity (340/380) correlates with the levels of intracellular K^+^.^29^

### Real-time PCR

Total RNA was isolated from cells using TriPure Isolation Reagent (Roche Applied Science, Milan, Italy) and reverse transcribed with the QuantiTect Reverse Transcription Kit (Qiagen, Milan, Italy). Real-time PCR was performed using SYBR Select Master Mix (Applied Biosystems, Monza, Italy) or Fluorocycles II SYBR Green Master mix (Euroclone) according to the manufacturer's instructions. Primers (designed by PRIMER 3 (v.0.4.0) and purchased from TIB MOLBIOL, Genoa, Italy)) and RT-PCR conditions are described in Supplementary Table S1. Target gene levels were normalized to that of GAPDH mRNA. Q-Gene program was used for analyzing gene expression.^51^

### Western blot analysis

Triton X-100 cell lysates were resolved on 12% SDS-PAGE and electrotransfered.^7, 50^ Filters were probed with 3ZD monoclonal antibody anti-IL-1*β* (IgG1 obtained from the National Cancer Institute Biological Resources Branch, Frederick, MD, USA), anti caspase-1 p10 rabbit polyclonal antibody (sc-514 purchased from Santa Cruz Biotechnology, DBA, Milan, Italy) and the mouse anti-*α*-tubulin monoclonal antibody (T6074 from Sigma).

### FACS analysis

Peripheral blood mononuclear cells (PBMCs) were stained with anti-v-ATPase (Novus Bio, Cambridge, UK), anti-CD14 or anti-CD3 (BD Pharmingen, Milan, Italy) Abs; mouse peritoneal macrophages were stained with anti-v-ATPase Ab (Proteintech, Manchester, UK). Samples were acquired using a FACS Canto II flow cytometer (Becton Dickinson, Mountain View, CA, USA) and data were analyzed with FCS express (De Novo Software, Glendale, CA, USA). Quadrant markers were set accordingly to unstained controls.

Relative fluorescence intensity was calculated as follows: mean fluorescence intensity after Ab staining/mean fluorescence intensity after isotype control staining.

### Mice

C57BL/6 J mice (6-–8 weeks) were from Envigo (Oxon, UK). Mice were handled according to the National Legislative Provisions (DL no. 116 of 27-01-1992 and no. 26/2014). The study was approved by the Regional Ethic committee of Liguria (project no. 344).

### Determination of the lethal dose of LPS and therapeutic dose of ESO

Different groups of mice were injected intravenously with LPS at different doses, from 6 to 15 mg/kg body weight in a volume of 0.1 ml of LPS-free PBS (phosphate**-**buffered saline). The dose of LPS found to induce mortality of >95% of mice within 72 h was 12.5 mg/kg body weight (not shown), and was used in all the experiments. A series of pilot experiments were carried out to establish the best therapeutic dose of ESO, which was found to be 12.5 mg/kg.

### LPS induced endotoxic shock and PPI treatment

Four groups of mice were treated. In three groups, ESO (12.5 mg/kg) was administered intraperitoneally in 0.2 ml of isotonic saline solution 30 min before or 30  or 90 min after injection of 12.5 mg/kg LPS. Untreated mice received intraperitoneally equal volume of saline solution. Blood was collected from the retro-orbital plexus under halothane anesthesia at 90 min or 4 h after LPS administration. Mice were monitored for clinical signs of endotoxemia and lethality every 4 h for the first 72 h, three times a day for 1 week and three times a week for 21 days. Body temperature was measured using a rectal thermometer and weight loss on a precision balance, at various times. No late deaths were observed in any of the experimental groups. A group of 12 mice was intraperitoneally injected with ESO, and a control group of 10 mice received the solvent as control. After 2 weeks all the mice were intravenously injected with LPS, blood was collected and mice monitored as above.

### LPS rechallenge of ESO-treated mice that survived LPS shock

Fifteen ESO-treated mice that had survived the first LPS shock and 10 naive C57BL/6 J mice (same control group used above) were rechallenged with LPS at the same dose after 3 weeks from the first injection. Blood was collected and mice were monitored as above.

### Zymosan-induced generalized inflammation

A group of mice that survived LPS+ESO and a group of naive C57BL/6 J mice were intraperitoneally injected with zymosan (1 g/kg in isotonic saline solution).^35^ Blood was collected and mice were monitored for clinical signs of endotoxemia and lethality as above.

### Thioglycollate-induced peritonitis

Two groups of three mice were injected intraperitoneally with 4% (wt/vol) of sterile DIFCO thioglycollate medium (1 ml per mouse; BD Biosciences, Milan, Italy).^36^ One group received ESO intraperitoneally 30 min before thioglycollate. Peritoneal exudate cells were obtained 4 h or 3 days later as described above. Total leukocyte cell counts were performed by hemocytometer, and differential cell counts were determined on Diff-Quick- (Polysciences Europe, Germany) stained cytospin preparations.

### Murine macrophages isolation and culture

Peritoneal macrophages were stimulated with 100 ng/ml LPS, 5 μg/ml R848, 20 μg/ml zymosan, 1 *μ*g/ml flagellin, 50 μg/ml poly(I:C) or 1 μg/ml PAM3 in RPMI-1640 with 5% FCS. In some experiments, macrophages were primed for 18 h with LPS, poly(I:C) or Pam(3)CSK(4) followed by ATP (5 mM, 30 min) or nigericin (20 *μ*M, 20 min). Cells were lysed for RNA extraction or western blot and supernatants were collected for ELISA.^7, 50^

### ELISA and transcription factor assays

Detection of cytokines in supernatants of human monocytes and mouse macrophages, or in the serum of mice was determined by specific kits (R&D Systems, Space, Milan, Italy). KC, MIP-2 and MCP-1 levels in serum and peritoneal lavage of mice were determined using Peprotech (DBA, Milan, Italy) ELISA Kits.

Analysis of p65 (NF-*κ*B), c-Fos and c-Jun (AP-1) levels in nuclear extracts from human monocytes was performed using Trans-AM Kits (Active Motif, Rixensart, Belgium) according to the manufacturer's instructions.

### Statistics

Statistical analysis was performed by using unpaired Student's *t*-test or one-way ANOVA test, followed by Bonferroni post-test, as appropriate. Survival rate was performed by Kaplan–Meier analysis and compared by the Mantel–Cox test. All tests were carried out using the GraphPad Prism (v.4.0, GraphPad Software, San Diego, CA, USA) and they were inferred as significant at *P*<0.05.

## Abbreviations

AP-1: activator protein-1
ATP: adenosine triphosphate
CAPS: cryopyrin-associated periodic syndrome
ESO: esomeprazole
H^+^/K^+^ ATPase: hydrogen potassium ATPase
IL: interleukin
LPS: lipopolysaccharide
MCP-1: monocyte chemoattractant protein-1
MIP-2: macrophage inflammatory protein 2
NF-*κ*B: nuclear factor-*κ*B
NLRP3: NOD-like receptor (NLR) family, pyrin domain containing 3
OME: Omeprazole
PAMP: pathogen-associated molecular pattern
PPI: proton pump inhibitor
P2X7: purinergic P2X, ligand-gated ion channel 7
TLR: Toll-like receptor
TNF-*α*: tumor necrosis factor-*α*
v-ATPase: vacuolar ATPase
