# Supplementary material for: Proton pump inhibitors protect mice from acute systemic inflammation and induce long-term cross-tolerance
Source: Cell Death Dis. 2016 Jul 21;7(7):e2304–. doi: 10.1038/cddis.2016.218 (PMC4973356; doi:10.1038/cddis.2016.218)
Supplement: Supplementary Information [file cddis2016218x1.pdf]

Supplementary Information

PROTON-PUMP INHIBITORS PROTECT MICE FROM ACUTE SYSTEMIC INFLAMMATION AND INDUCE LONG TERM CROSS-TOLERANCE ***(Balza et al.)***

Supplementary Table S1. Primers and conditions for Real Time PCR

Gene (ID)	Primer sequence	Primer concentrations (μM/L)	Amplicon size (bp)	Ta (°C)
h-*IL-1β* (3553)	F 5' TCCAGGGACAGGATATGGAG 3'	0,25	133	60
	R 5' TCTTTCAACACGCAGGACAG 3'	0,25		
h-*TNF-α* (7124)	F 5' CAGGGACCTCTCTCTAAT 3'	0,25	98	58
	R 5' GAGGGTTTGCTACAACAT 3'	0,25		
h-*GAPDH* (2597)	F 5' GAAGGTGAAGGTCGGAGTC 3'	0,2	155	60
	R 5' CATGGGTGGAATCATATTGGAA 3'	0,2		
m-*P2rx7* (18439)	F 5' TCCACCCTGTCCTACTTTGG 3'	0,25	116	60
	R 5' CAGGGCTCACAGCACTTACA 3'	0,25		
m-*ATP4b* (11945)	F 5' GGTAACCTTGAGACCGGACG 3'	0,2	149	60
	R 5' AGTTGATGCTGTCCTGCTGG 3'	0,2		
m-*GAPDH* (14433)	F 5' ATGGCCTTCCGTGTTCCTAC 3'	0,2	100	60
	R 5' GCTTCACCACCTTCTTGATGTC 3'	0,2		

Gene ID from www.ncbi.nlm.nih.gov/gene; : h : human; m: mouse; F: forward; R: reverse; Ta: annealing temperature; *IL-1β:* Interleukin 1 beta; *TNF-α* : Tumor Necrosis Factor; *GAPDH*: Glyceraldehyde-3-Phosphate Dehydrogenase; *P2rx7:* purinergic receptor P2X, ligand-gated ion channel, 7; *ATP4b* : ATPase, H+/K+ exchanging, beta polypeptide.
