# Supplementary figures and images for: Proton pump inhibitors protect mice from acute systemic inflammation and induce long-term cross-tolerance
Source: Cell Death Dis. 2016 Jul 21;7(7):e2304–. doi: 10.1038/cddis.2016.218 (PMC4973356; doi:10.1038/cddis.2016.218)

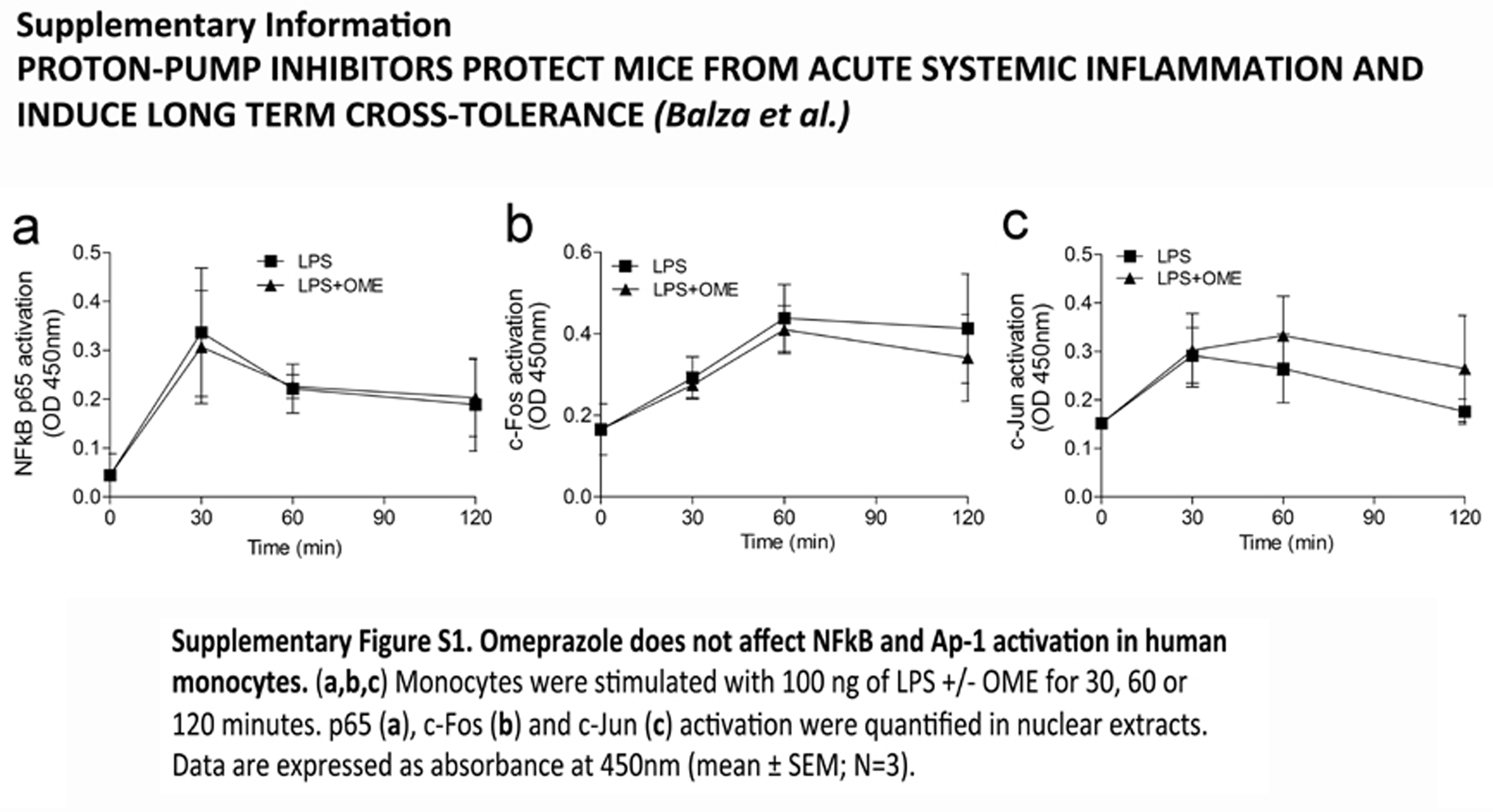

Supplement: Supplementary Figure 1 [file cddis2016218x2.tif]

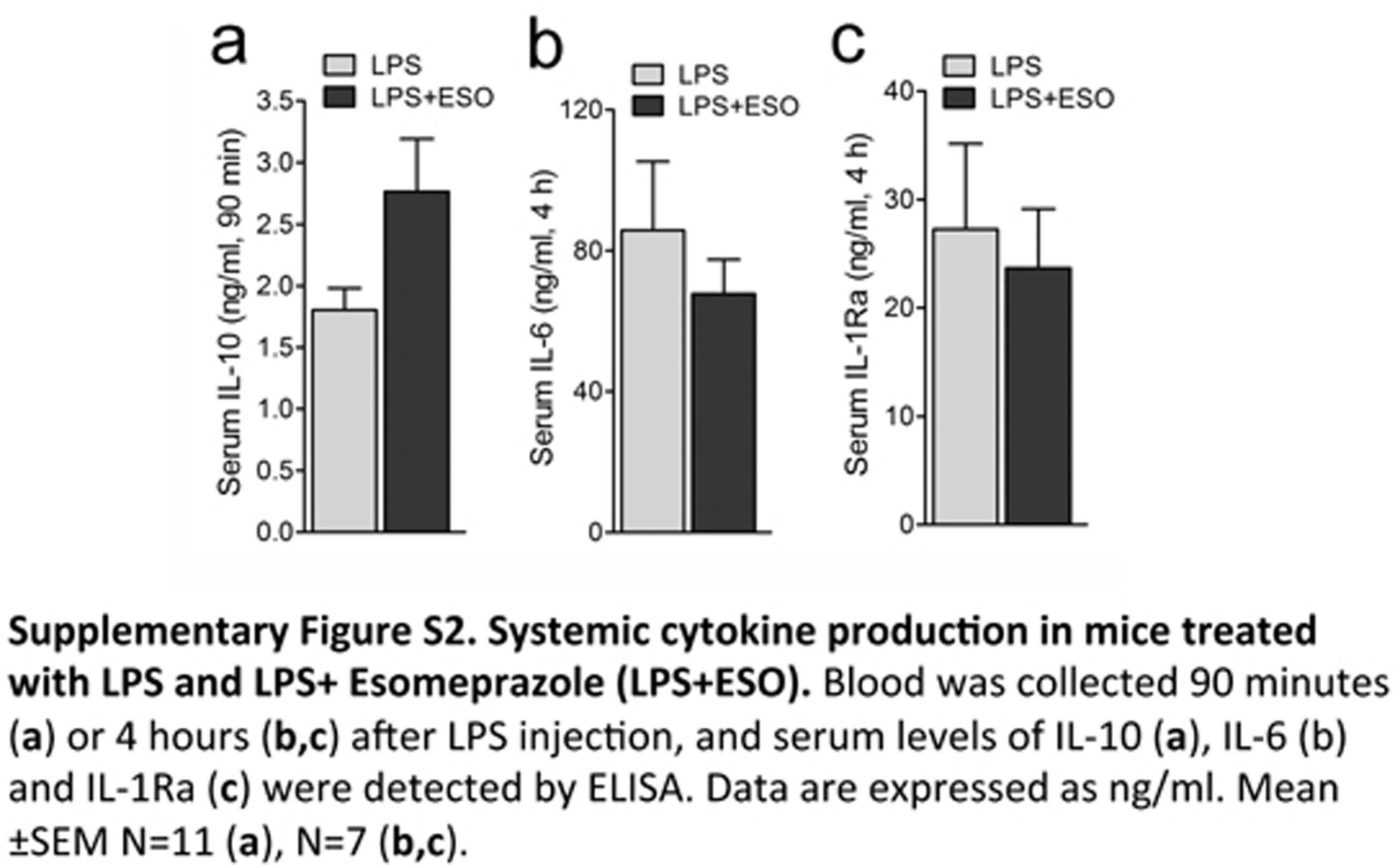

Supplement: Supplementary Figure 2 [file cddis2016218x3.tif]

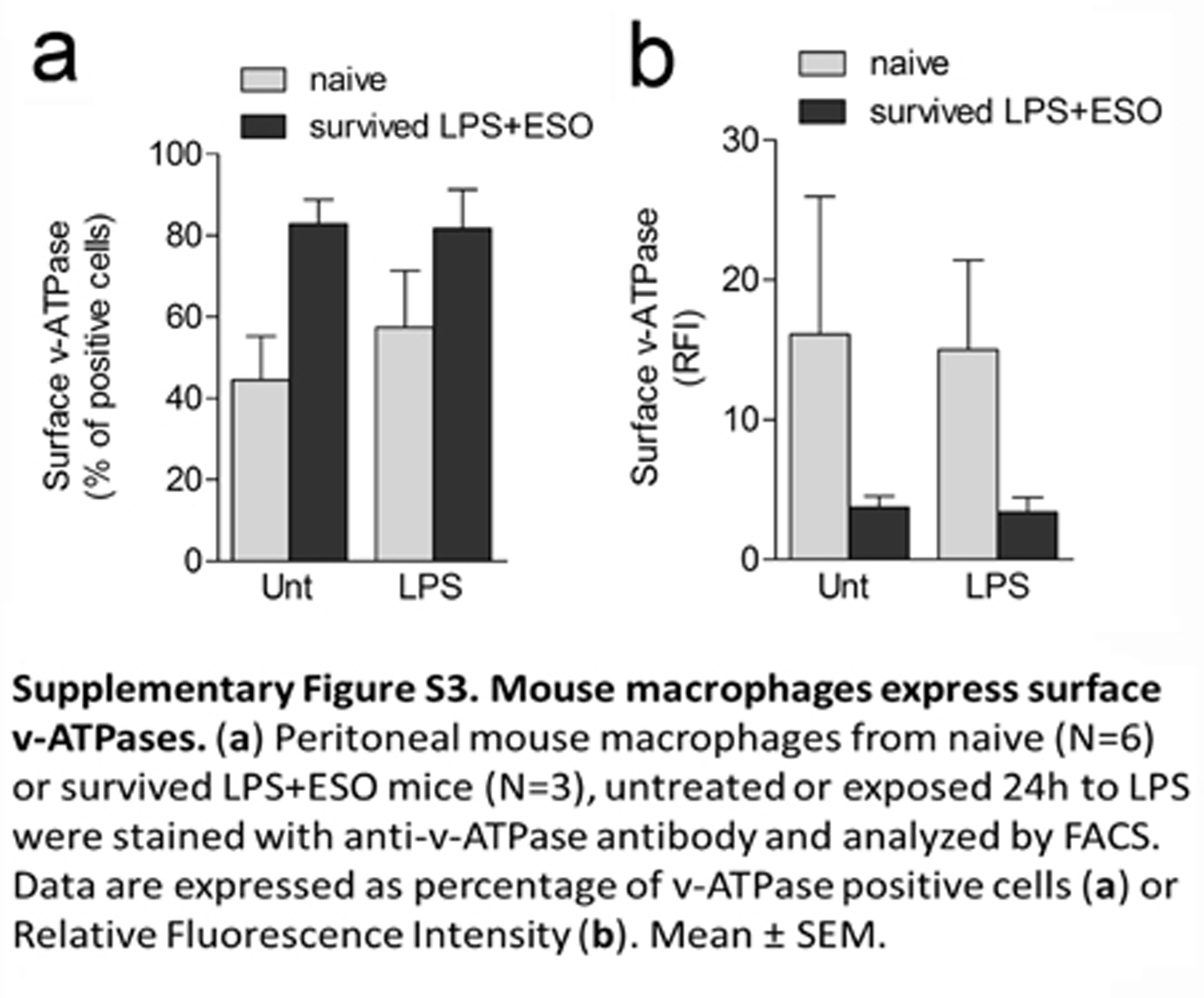

Supplement: Supplementary Figure 3 [file cddis2016218x4.tif]
